# “We want everything in a one-stop shop”: acceptability and feasibility of PrEP and buprenorphine implementation with mobile syringe services for Black people who inject drugs

**DOI:** 10.1186/s12954-022-00721-6

**Published:** 2022-12-03

**Authors:** Tyler S. Bartholomew, Barbara Andraka-Cristou, Rachel K. Totaram, Shana Harris, Susanne Doblecki-Lewis, Lily Ostrer, David P. Serota, David W. Forrest, Teresa A. Chueng, Edward Suarez, Hansel E. Tookes

**Affiliations:** 1 Division of Health Services Research and Policy, Department of Public Health Sciences, Miller School of Medicine, University of Miami, 1120 NW 14th St., #1020, Miami, FL 33136 USA; 2 Department of Health Management and Informatics, University of Central Florida, Orlando, FL USA; 3 Department of Internal Medicine, University of Central Florida, Orlando, FL USA; 4 Department of Anthropology, University of Central Florida, Orlando, FL USA; 5 Division of Infectious Diseases, Department of Medicine, University of Miami Miller School of Medicine, Miami, FL USA; 6 Department of Anthropology, College of Arts and Sciences, University of Miami, Miami, FL USA; 7 Department of Psychiatry and Behavioral Sciences, University of Miami Miller School of Medicine, Miami, FL USA

**Keywords:** PrEP, Medications for opioid use disorder, Syringe services program, Black people who inject drugs

## Abstract

**Introduction:**

A recent surge in HIV outbreaks, driven by the opioid and stimulant use crises, has destabilized our progress toward targets set forth by *Ending the HIV Epidemic: A Plan for America* for the high-priority community of people who inject drugs (PWID), particularly Black PWID.

**Methods:**

In order to ascertain the acceptability and feasibility of using a mobile syringe services program (SSP) for comprehensive HIV prevention via PrEP and medications for opioid use disorder (MOUD), our mixed methods approach included a quantitative assessment and semi-structured qualitative interviews with Black PWID (*n* = 30) in Miami-Dade County who were actively engaged in mobile syringe services.

**Results:**

Participants felt that delivery of MOUD and PrEP at a mobile SSP would be both feasible and acceptable, helping to address transportation, cost, and stigma barriers common within traditional healthcare settings. Participants preferred staff who are compassionate and nonjudgmental and have lived experience.

**Conclusions:**

A mobile harm reduction setting could be an effective venue for delivering comprehensive HIV prevention services to Black PWID, a community that experiences significant barriers to care via marginalization and racism in a fragmented healthcare system.

## Introduction

The goals of the *Ending the HIV Epidemic (EHE)* initiative in the United States (US) remain elusive for the key population of people who inject drugs (PWID), with 11% of new HIV infections attributable to injection drug use (IDU) [1]. EHE goals will be unattainable without community-level harm reduction and targeted interventions, particularly in the face of a growing number of IDU-associated HIV outbreaks [2–7]. HIV pre-exposure prophylaxis (PrEP) and syringe services programs (SSPs) are cornerstone evidence-based prevention interventions of the “Protect*”* pillar of EHE [8, 9]. Yet, implementation and scale-up of PrEP and SSPs remain low, especially in the US South [10, 11], further exacerbating racial and ethnic disparities in new HIV infections. For example, in Miami, Florida, the city with the highest rate of HIV infection in the nation, Black race is the strongest predictor of HIV infection among PWID [12].

While HIV incidence decreased among PWID between 2004 and 2015, the recent surge in HIV outbreaks associated with IDU has destabilized our progress toward EHE targets for this high-priority community, particularly Black PWID, who comprise 27% of new HIV diagnoses due to IDU [1–5, 7, 13]. The increasing frequency of outbreaks has been fueled by the convergence of the opioid and stimulant use disorder crises in the US [14–19], which has led to significantly more people injecting drugs than ever before [20]. With over 108,000 people losing their lives to preventable overdose in 2021 [21], comprehensive HIV prevention has become progressively more complicated. This complex syndemic of HIV, substance use, and overdose has been exacerbated due to COVID-19, with curtailed essential harm reduction services [22, 23] including reductions in HIV/hepatitis C virus (HCV) testing and treatment [24–26], decreased access to medications for opioid use disorder (MOUD) [27–29], and disruptions in the HIV care and PrEP continuum [30].

PrEP remains an understudied and underutilized HIV prevention strategy for PWID in general, and Black PWID in particular. The Bangkok Tenofovir Study showed a 49% decrease in HIV incidence in PWID among those taking daily tenofovir compared to placebo and revealed significant interest among PWID in continuing PrEP after the trial [31, 32]. Unfortunately, there has been a large gap in research about PrEP implementation among PWID since the Bangkok Tenofovir Study was completed in 2010 [33]. A recent systematic literature review showed that while there is high awareness and willingness to use PrEP among PWID, uptake was only 0–3% [34]. Structural and social barriers to PrEP can impede access to groups at highest risk (e.g., Black PWID) and limit its impact. Barriers to PrEP, including cost, transportation, health literacy, stigma, and immigration status, can create disparities in PrEP engagement that particularly impact people of color and the drug-using community [35]. New delivery models are essential to increase PrEP uptake in the face of competing priorities in this marginalized community [36].

Integrated treatment for HIV and opioid use disorder (OUD) is essential to improve health of patients with OUD [37], and retention in OUD treatment has been shown to predict long-term HIV viral suppression [38–41]. Buprenorphine, an evidence-based MOUD, has long been shown to improve outcomes in individuals with OUD [42], but racial disparities in access to buprenorphine treatment are also salient [43]. An escalation in the opioid epidemic has recently occurred in Black communities, with opioid deaths having risen 43% among Black people with OUD over the past 5 years compared to a 22% increase among White people with OUD over the same time period [44–46]. Recent meta-analyses indicate that buprenorphine decreases the risk of all-cause mortality for people with OUD by approximately 50% [47]. However, despite increases in the number of people in the US receiving buprenorphine in recent years, numerous studies indicate significant racial/ethnic disparities in buprenorphine treatment access [20, 43, 48–53]; Black patients with OUD are 77% less likely to receive buprenorphine prescriptions compared with White patients [43] as the result of structural racism within the healthcare system.

Low-barrier buprenorphine treatment initiation approaches could help increase access for PWID, including people of color. For example, in New Jersey, mobile medical units have reported success enrolling more Black PWID, persons experiencing homelessness, and uninsured patients compared to fixed-site opioid treatment programs (i.e., programs that offer methadone, another effective MOUD) [54]. Similarly, at our IDEA Miami SSP in Miami, Florida, we have seen significantly higher rates of engagement in syringe services among Black PWID, homeless PWID, and PWID with HCV at our mobile unit locations compared to our fixed site, suggesting a mobile strategy may be necessary to increase access to care for this high-priority community [55]. In pursuit of implementation strategies to mitigate these health disparities buttressed by structural racism in the US South, this study seeks to ascertain the acceptability and feasibility of using a mobile harm reduction approach to deliver PrEP and MOUD to Black PWID, a long overlooked and understudied community.

## Methods

### Study setting

We conducted semi-structured qualitative interviews with Black PWID in Miami-Dade County, the United States, who were actively engaged in mobile SSP services. Participants were recruited from the mobile SSP satellite site in a predominately Black community of Miami-Dade County.

### Data collection

Inclusion criteria for this study were age 18 years or older, HIV-negative serostatus, injection of an opioid in the previous 30 days, and self-identified Black race. Data were collected through convenience sampling between June and August 2020 at the time of receipt of harm reduction services. Recruitment occurred across multiple locations that the mobile SSP targets within predominately Black communities. All interviewers received training on qualitative methods and how to conduct semi-structured interviews, and all interviews were conducted in a private, face-to-face meeting. Interviews lasted approximately 25–30 min. The lead qualitative researcher (DWF) holds a PhD in Anthropology, is the program director of the SSP, and supervised the qualitative interviews.

Interview topics included the following: PrEP awareness; current/previous PrEP use; perceived PrEP effectiveness in prevention of HIV infection; MOUD experiences; perceptions of effectiveness, acceptability, and feasibility of receiving PrEP and MOUD services on the mobile SSP unit; and previous experiences (barriers/facilitators) in accessing PrEP and MOUD services. Qualitative interviews were audio-recorded and transcribed verbatim in English.

Additionally, at the time of the interview, participants completed a short quantitative background/demographic assessment, which was recorded on a tablet computer using REDCap [56]. This short assessment included questions about demographics (e.g., age, biological sex, educational attainment, housing status), self-reported PrEP and MOUD knowledge, and previous experience with PrEP and MOUD. Participants were compensated $25 for their time and participation.

### Data analysis

Descriptive statistics from the quantitative assessment were reported as medians and interquartile ranges for continuous variables and frequency distributions for categorical variables. All quantitative analyses were performed in SAS statistical software (Version 9.4; SAS Institute, Cary, NC). Qualitative data were analyzed by BAC, SH, and RKT, all of whom are health service researchers with extensive qualitative analysis experience. Interview data were analyzed using iterative categorization, a systematic deductive–inductive approach to analyzing qualitative data, developed for the substance use disorder (SUD) research field, using the following steps [57]: First, a codebook was created based on the research questions and a preliminary review of interview data. Second, the qualitative researchers each independently coded six interview transcripts in Dedoose mixed method software [58], deductively applying codes to meaningful data. Next, the researchers met to discuss coding discrepancies, revising the codebook as needed to reflect inductively identified new categories of data. Multiple codes could be applied to a single excerpt. Since few discrepancies in coding existed for the first six interviews, the remainder of the transcripts were divided between the researchers to code independently.

After coding was completed, coded excerpts were exported to a Microsoft Excel document, with one sheet per code. Researchers labeled each excerpt with summary statements (e.g., one paragraph of text might be summarized as “participant felt employees with lived experience are trustworthy”). Researchers then independently examined across all excerpt labels and created a code summary based on consistencies/inconsistencies in the excerpt labels. Researchers met to compare code summaries, negotiating any differences until a final code summary was created. This process was repeated for each code. Finally, the qualitative researchers independently reviewed across all code summaries to identify overarching themes. Researchers met to compare themes, negotiating until they agreed on final themes, which are described below. These final themes were presented to the larger research group for additional feedback. Authors referred to the Consolidated Criteria for Reporting Qualitative Research (COREQ) checklist to guide the reporting of study methods and findings (see “Appendix A”) [59].

## Results

### Participant characteristics

Thirty total participants (*n* = 30) completed the semi-structured interview and quantitative assessment. Participant characteristics are presented in Table 1. All participants were actively using SSP services. The median age of the sample was 53; 80% were non-Hispanic Black; 93% were male; and 57% were homeless.

**Table 1:** Socio-demographic, drug use, and PrEP/MOUD knowledge, awareness, and interest among Black PWID (*N* = 30)

Characteristic	Total *n* (%)
Age (median, IQR)	53 (32–62)
Biological sex (*n*, %)	
Male	28 (93.3)
Female	2 (6.7)
Race/ethnicity (*n*, %)	
Non-Hispanic black	24 (80.0)
Hispanic black	4 (13.3)
Refused	2 (6.7)
Annual income (*n*, %)	
< $14,999	25 (92.6)
> $15,000	2 (7.4)
Insurance status (*n*, %)	
Medicaid	7 (23.3)
Private	3 (10.0)
No insurance	19 (63.3)
Housing status (*n*, %)	
Currently experiencing homelessness	17 (56.7)
Not experiencing homelessness	13 (43.3)
Substances injected in previous 30 days (*n*, %)	
Heroin	25 (83.3)
Cocaine	15 (50.0)
Methamphetamine	7 (23.3)
Crack-cocaine	8 (26.7)
Speedball	10 (33.3)
Fentanyl/carfentanil	16 (53.3)
PrEP assessment	
Ever heard of PrEP (*n*, %)	
Yes	18 (60.0)
No	12 (40.0)
Ever taken PrEP (*n*, %)	
Yes	2 (6.7)
No	28 (93.3)
Would you like to receive information about PrEP? (*n*, %)	
Yes	20 (66.7)
No	10 (33.3)
In what way(s) would you prefer to receive more information about PrEP? (*n*, %)	
Brochures	2 (6.7)
Videos	4 (13.3)
Lectures	0 (0.0)
Focus groups	3 (10.0)
At the SSP	7 (23.3)
From a peer currently taking PrEP	18 (60.0)
How interested are you in receiving PrEP for free here on the SSP mobile unit? (*n*, %)	
Not interested at all	6 (20.0)
Somewhat interested	16 (53.3)
Very interested	8 (26.7)
MOUD assessment	
Ever heard of MOUD	
Yes	29 (96.7)
No	1 (3.3)
Ever taken a MOUD (*n*, %)	
Yes	23 (76.7)
No	7 (23.3)
Currently taking a MOUD (*n* = 23) (*n*, %)	
Yes	3 (13.0)
No	20 (87.0)
MOUD used (*n* = 23) (*n*, %)	
Methadone	6 (26.1)
Buprenorphine	16 (69.6)
Extended-release naltrexone	1 (4.3)
Would you like to receive information about buprenorphine? (*n*, %)	
Yes	25 (83.3)
No	5 (16.7)
In what way(s) would you prefer to receive more information about buprenorphine? (*n*, %)	
Brochures	3 (10.0)
Videos	3 (10.0)
Lectures	0 (0.0)
Focus Groups	1 (3.3)
At the SSP	11 (36.7)
From a peer currently on MOUD	18 (60.0)
How interested are you in receiving buprenorphine for free here on the SSP mobile unit? (*n*, %)	
Not at all interested	4 (13.3)
Somewhat interested	10 (33.3)
Very interested	16 (53.3)

### PrEP/MOUD knowledge, preference, and interest

Most participants reported using either heroin (83%) or fentanyl (53%) in the previous 30 days. The majority (93.3%) had never taken PrEP but knew of it (60%); however, most participants (97.7%) had heard of a MOUD and had been prescribed a form of MOUD previously (77%). In addition, most participants (60%) expressed preference in receiving information about PrEP and MOUD from a peer currently on either medication. Most participants expressed interest in receiving PrEP (80%) and/or MOUD (87%) services at the mobile SSP.

### Qualitative interview results

#### Theme 1: participants felt an SSP mobile unit would be a safe, confidential, and comfortable location for receiving MOUD and/or PrEP

Almost all participants felt people already accessing other SSP services would be comfortable accessing PrEP and/or MOUD through the SSP. Participants felt that the SSP would work well as a “one-stop shop” for multiple services. However, participants noted that SSP outreach staff would need to inform the broader community of the additional services being offered at the SSP besides traditional harm reduction services (i.e., syringe exchange, naloxone distribution). The SSP was described as a comfortable place because it was specifically designed to meet the needs of people who use drugs. For example, Participant 16 said, “Of course [I’d be comfortable] because [the SSP] is designed just for us users.”

Participants also noted that the SSP was already known as a “safe place” with caring employees who were non-stigmatizing and respected patient confidentiality—all important health service delivery factors for SUD/HIV services. Participant 9 cautioned, however, that people cannot feel “safe” in the SSP if police officers are nearby: “[The SSP should be] secure, as in the cops don’t hang out around here. I think that’s a continual concern for all of us in the community.” Only a few participants said they would feel uncomfortable obtaining MOUD or PrEP from the SSP, citing concerns about stigma related to their personal hygiene or HIV status.

One participant felt the SSP’s “open door” policy would allow people to “just come in here and see what it’s about,” including learning about PrEP or MOUD, before committing to starting treatment. Furthermore, participants noted that people might feel more comfortable obtaining MOUD and/or PrEP from the SSP if they already knew other people obtaining similar services through the SSP.

#### Theme 2: the SSP mobile unit is more accessible than other SUD treatment/HIV prevention programs, and accessibility could be further enhanced through strategic locations and open hours

The mobile unit was considered more accessible than traditional SUD treatment and HIV prevention programs, especially considering the frequency of necessary follow-up appointments and laboratory work. Several participants believed people needing MOUD or PrEP might lack cars or public transportation access. Participants felt that a mobile unit within walking distance could address transportation barriers, but only if people were aware of the location/times of mobile unit services. Participants also implicitly described the mobile unit as a person-centered delivery structure because the unit came to them, meeting them “where they are at.”

To further increase accessibility, participants felt the mobile unit should specifically visit locations where drug use is known to be common. Participants provided the names of specific streets/intersections to researchers, as well as general types of locations, including hotels/motels, bus stops, areas where free food is provided, gas stations, under bridges, and parks. Most participants felt the SSP mobile unit should be available every day of the week, including weekends. Participants also overwhelmingly preferred that the SSP mobile unit visit their location multiple times per day (e.g., morning, afternoon, and night), as opposed to once per day because times of drug use and sleep vary.

#### Theme 3: participants desired SSP staff who were caring, nonjudgmental, approachable, and had lived drug use experience

When asked what characteristics they would like to see in SSP staff, participants routinely described caring and nonjudgmental personalities. A few noted that staff should also be good humored, as demonstrated by terms such as “funny,” “friendly,” and “sociable.” Additionally, several participants felt staff should be honest and not “take bullshit” from SSP participants. Participant 13 noted, “[People] don't need to be preached to. Just need to have a good heart and know how to relate to people.” Participants also felt SSP staff should be approachable. For instance, while many participants said they were indifferent to SSP staff clothing, several mentioned that staff should wear casual clothing as opposed to scrubs, so as not to imply that staff are “better than” participants. One participant said scrubs would “scare” him away, and another participant explained that he would not have approached the SSP but for the casual clothing of staff.

Most participants explained that they did not care about the SSP staff members’ race or ethnicity, but a few strongly desired at least some Black staff members. For example, Participant 18 said, “[Staff need] not only experience with injection, but [also] that experience with the stigmatization that goes with [being] a Black individual." Almost all participants felt SSP staff should have lived experience, with several noting that only lived experience could provide deep knowledge about drug use. For example, Participant 22 explained, “You can get certain things out of a book, but if you ain’t experienced it, you really don’t know what you gettin’ into.” Another participant explained that many people who use drugs do not trust others (e.g., due to fear of criminal prosecution), and staff lived experience implicitly suggests the staff are relatable and trustworthy. For example, Participant 13 said, “As much as possible some maybe ex-addicts themselves that can relate.” A few participants said lived experience was not necessary, so long as staff are knowledgeable about drug use. Among participants who desired staff with lived experience, several implied the importance of experience with injecting by using phrases like “injection experience” rather than merely “drug experience.” Additionally, through phrases such as “ex-addict,” a few participants implied preference for staff with lived experience with SUD who are in recovery.

## Discussion

PWID experience substantial discrimination, stigma, and considerable social disadvantage when accessing healthcare in traditional settings in the US, leading to poorer health outcomes than comparable populations who do not inject drugs [60–68]. Previous research has demonstrated the extensive multilevel barriers PWID face when accessing health services [69], including PrEP and MOUD, and has suggested that traditional healthcare systems are “risk” environments [70]. Since SSPs do not mimic the more formal built environment of most healthcare settings, they could be ideally situated to provide HIV prevention and MOUD treatment to those who are alienated from other healthcare environments. In this study of thirty participants of the IDEA Miami SSP in Miami, Black PWID found the idea of PrEP and MOUD services delivered through a mobile SSP acceptable and emphasized that this setting has fewer barriers to access than traditional healthcare settings by meeting clients” where they are” [71, 72]. Participants also noted that mobile SSP units are particularly accessible by helping to limit transportation barriers, especially if mobile SSP units visit areas frequently populated by PWID during a wide range of hours, all days of the week.

Importantly, our study conceptualizes accessibility of SSP services broadly, not just with respect to time and place of services, but also with respect to staff behavior and other service factors that increase client comfort receiving services. To our knowledge, SSPs typically strive to employ nonjudgmental and compassionate staff, but our findings suggest that even clothing of staff can impact client comfort. For example, while most participants in our study said they were indifferent to staff appearance, a few noted that staff in scrubs or non-casual clothing would make them feel uncomfortable receiving services. We are unaware of other studies discussing the role of SSP staff clothing in increasing client comfort. Additionally, our study found that people outside of the SSP (e.g., local police) could hinder comfort accessing services in the SSP. Given the tenuous historical relationship between police and Black PWID in the US, one potential benefit of mobile SSP services (as compared to fixed SSP locations) is that they can move to different locations in response to police presence.

As indicated by our qualitative interviews, PWID prefer to access healthcare in a highly accessible (e.g., mobile) “one-stop shop,” and SSPs could provide that optimal venue. Other studies have also noted a wide range of services that can feasibly be provided at SSPs, including HIV/HCV testing [73], linkage to substance use treatment [74, 75], HIV [76] and HCV treatment [77], on-site MOUD [78], on-site PrEP [79], overdose prevention through naloxone distribution [80], and medical services including wound care and general primary care [81]. Prior research suggests that MOUD use improves HIV outcomes—an additional argument for increasing access to MOUD among people living with HIV or at risk of HIV. For example, MOUD has been shown to predict HIV viral suppression and to increase retention on HIV treatment [38–41].

Developing novel approaches to increase uptake, initiation, and use of PrEP is critical, as prior research shows very low utilization among PWID (0–3%) [34, 82–85], despite willingness that ranged in prior studies from low-to-high (23–79%) [86–88]. For example, our previous report found that 57.2% of PWID at the IDEA Miami SSP were interested in initiating PrEP [86], suggesting that low uptake is due to issues other than lack of interest. In pursuit of innovative implementation strategies, there have been recent reports of telehealth implementation for access to MOUD at SSPs during the COVID-19 pandemic [89–91] and limited research on a comprehensive, integrated, evidence-based, feasible intervention to address HIV prevention and substance use among PWID [92–94]. There is an urgent need for hybrid implementation-effectiveness trials to build upon the foundational effectiveness of harm reduction strategies and examine the effectiveness of innovative delivery models (e.g. telehealth technology, mobile units) to bring comprehensive HIV prevention to the people where they are. Our study is unique by focusing on the perspectives and experiences of Black PWID. Black PWID face overlapping drug use stigma and racism [43, 48], as well as stigma related to HIV. Despite both the precipitous rise in overdose deaths among Black people with OUD and significantly higher risk of HIV transmission in this community, MOUD and PrEP are more available to White PWID [1, 44–46], and scientific and popular discourse focus disproportionately on White PWID [53]. Our qualitative data suggest that Black PWID have a strong preference for staff with lived experience. Furthermore, while most study participants did not feel race/ethnicity of SSP staff mattered, a few felt strongly that SSP staff race/ethnicity should mirror that of the community they serve.

### Limitations

The current study is subject to several limitations. Our study assessed acceptability and feasibility of offering PrEP and MOUD services for Black PWID in a mobile SSP in a single large city, which may not be generalizable to other settings. Additionally, most study participants were male, and these findings may not apply to the broader Black PWID community. Given the low number of women, our survey may not have uncovered different barriers and facilitators to accessing services and would not reveal the importance of addressing gender-specific needs, including sexual violence experienced by women [79]. Also, participants in this study utilized mobile services of the SSP, rather than the fixed site location, which may limit generalizability to fixed-site harm reduction settings. However, we have previously found that Black PWID are more likely to engage in our mobile clinic services [55]. Finally, our data collection coincided with the onset of COVID-19 pandemic mitigation restrictions, which may have limited participation and altered preferences due to more limited accessibility of transportation and other services.

## Conclusion

There is an urgent need for innovative, efficacious, scalable, and community-driven models of comprehensive HIV services for the Black PWID community, rooted in non-stigmatizing venues to overcome overlapping discrimination as well as logistical challenges [53]. We found that Black PWID who utilized our urban mobile SSP believed provision of MOUD and PrEP at the mobile SSP would be both feasible and acceptable, particularly because MOUD and PrEP in the mobile setting would help address transportation, cost, and stigma that are barriers within traditional healthcare settings. Our findings also indicate potential benefits of a mobile service delivery approach in addition to a fixed site SSP which could have added benefits by providing multiple, strategic locations and more flexibility in hours of operation. We highlight the need for people who provide MOUD and PrEP services at mobile SSPs to be compassionate, nonjudgmental, and to have lived experience with SUD. A mobile harm reduction setting could be an effective model of delivering comprehensive HIV prevention services to Black PWID, a community that experiences significant barriers to care via marginalization and racism in a fragmented healthcare system.

## Data Availability

Data are available by the corresponding author upon reasonable request.

## Appendix A

See Table 2.

**Table 2:** Consolidated criteria for reporting qualitative studies (COREQ): 32-item checklist

No. item	Guide questions/description	Reported on page #
*Domain 1: research team and reflexivity*		
Personal characteristics		
1. Interviewer/facilitator	Which author/s conducted the interview or focus group?	Results
2. Credentials	What were the researcher’s credentials? E.g., PhD, MD	Methods
3. Occupation	What was their occupation at the time of the study?	Methods
4. Gender	Was the researcher male or female?	N/A
5. Experience and training	What experience or training did the researcher have?	Methods
Relationship with participants		
6. Relationship established	Was a relationship established prior to study commencement?	N/A
7. Participant knowledge of the interviewer	What did the participants know about the researcher? e.g., personal goals, reasons for doing the research	N/A
8. Interviewer characteristics	What characteristics were reported about the interviewer/facilitator? e.g., Bias, assumptions, reasons and interests in the research topic	Methods
*Domain 2: study design*		
Theoretical framework		
9. Methodological orientation and Theory	What methodological orientation was stated to underpin the study? e.g., grounded theory, discourse analysis, ethnography, phenomenology, content analysis	Methods
Participant selection		
10. Sampling	How were participants selected? e.g., purposive, convenience, consecutive, snowball	Methods
11. Method of approach	How were participants approached? e.g., face-to-face, telephone, mail, email	Methods
12. Sample size	How many participants were in the study?	Results
13. Non-participation	How many people refused to participate or dropped out? Reasons?	Methods
Setting		
14. Setting of data collection	Where was the data collected? e.g., home, clinic, workplace	Methods
15. Presence of non-participants	Was anyone else present besides the participants and researchers?	Results
16. Description of sample	What are the important characteristics of the sample? e.g., demographic data, date	Results
Data collection		
17. Interview guide	Were questions, prompts, guides provided by the authors? Was it pilot tested?	Methods
18. Repeat interviews	Were repeat interviews carried out? If yes, how many?	N/A
19. Audio/visual recording	Did the research use audio or visual recording to collect the data?	Methods
20. Field notes	Were field notes made during and/or after the interview or focus group?	Methods
21. Duration	What was the duration of the interviews or focus group?	Methods
22. Data saturation	Was data saturation discussed?	Methods
23. Transcripts returned	Were transcripts returned to participants for comment and/or correction?	N/A
*Domain 3: analysis and findings*		
Data analysis		
24. Number of data coders	How many data coders coded the data?	Methods
25. Description of the coding tree	Did authors provide a description of the coding tree?	N/A
26. Derivation of themes	Were themes identified in advance or derived from the data?	Methods
27. Software	What software, if applicable, was used to manage the data?	Dedoose
28. Participant checking	Did participants provide feedback on the findings?	Strengths and limitations
Reporting		
29. Quotations presented	Were participant quotations presented to illustrate the themes/findings? Was each quotation identified? e.g., participant number	Results
30. Data and findings consistent	Was there consistency between the data presented and the findings?	Relationship to existing knowledge
31. Clarity of major themes	Were major themes clearly presented in the findings?	Results
32. Clarity of minor themes	Is there a description of diverse cases or discussion of minor themes?	Discussion
